# Biomass residues improve soil chemical and biological properties reestablishing native species in an exposed subsoil in Brazilian Cerrado

**DOI:** 10.1371/journal.pone.0270215

**Published:** 2022-06-27

**Authors:** Thaís Soto Boni, Engil Isadora Pujol Pereira, Adriana Avelino Santos, Ana Maria Rodrigues Cassiolato, Kátia Luciene Maltoni

**Affiliations:** 1 Department of Plant Protection, Rural Engineering and Soils, School of Engineering, São Paulo State University (UNESP), Ilha Solteira, São Paulo, Brazil; 2 School of Earth, Environmental, and Marine Sciences, The University of Texas Rio Grande Valley, Edinburg, Texas, United States of America; UNSW SYDNEY, AUSTRALIA

## Abstract

Revegetation of exposed sub-soil, while a desirable strategy in the recovery processes, often fails due to extreme soil chemical properties, such as low organic matter and pH levels inhospitable to biological activities such as nutrients cycling and plant establishment. This is the case for approximately 800 ha of the Cerrado biome in Brazil, where erecting the embankment of a hydroelectric dam in the 1960’s stripped vegetation, soil, and subsoil layers thereby distorting the soil properties. This work evaluates the effectiveness of restoration management (RM) treatments, to restore the soil quality, including biological activity and chemical attributes. In a factorial scheme, RM treatments include the addition of organic residue from aquatic macrophytes (AM) at 3 rates (0, 16 and 32 t ha^-1^), combined with ash from sugar cane bagasse of agroindustrial origin (BA) at 4 rates (0, 15, 30 and 45 t ha^-1^). RM samples contrasted samples collected from undisturbed Cerrado (CER) as well as a degraded area without intervention (DAWI). The mechanized RM plots received amendments and reforestation of 10 Cerrado native tree species. After 5 years, vegetation covered up to 60% of the surface in RM treatments receiving AM32 + BA45. AM and BA residues promoted height increases in the introduced plants. All RM treatments promoted lower levels of Al^3+^ than DAWI and CER. The combination of AM32 over the rates of incorporated ash increased soil pH and K values similarly to CER. Microbial-related variables, such as microbial biomass-C was the largest in CER, followed by the RM treatments, and the lowest in DAWI. The microbial quotient was no different between CER and RM treatments. The addition of residues such as AM and BA increased the vegetation covered, improved chemical and microbiological indicators. Thus, the residues used aided the recovery process of intensely degraded soils in the Cerrado area.

## Introduction

With an area of approximately two million square kilometers, the Cerrado biome forms the second largest biome in South America. Anthropic threats endangering the biome’s species richness and endemism placed Cerrado on the list of critical areas for biodiversity conservation worldwide [1]. Since the 1960s, approximately half of all Cerrado area became domesticated for agriculture, livestock as well as infrastructure for transport and energy production, promoting a severe degradation of this ecosystem [2]. These land use changes led to the loss of soil quality and its functional activities, resulting in the loss of ecosystem services, such as carbon storage, nutrient cycling, and soil formation [3].

Also in the 1960s, the construction of the Ilha Solteira Hydroelectric Power Plant (HPP-ISA) fueled civil growth in the states of São Paulo and Mato Grosso do Sul, generating significant extensions of anthropogenic areas (approximately 8,000,000 m^2^) [4]. Building the waterways and other structures for the HPP removed native Cerrado vegetation as well as soil extending as much as 12 m deep from the surface [5, 6]. The elimination of these materials left a geological residue that resembles mined areas of very low resilience, as they are devoid of edaphic attributes that enable spontaneous plant colonization [7].

The vegetation removal and the stripping, excavation, and transportation of the soil have different effects on soil physical, chemical, and biological properties [8]. Rebuild the soil after a degradation process is an essential factor for a successful restoration process [9]. Several techniques have been used to restore and control soil losses and water runoffs, such as revegetation, which helps in recovering organic matter, restructuring the degraded soil [10], and the use of organic soil amendments to boost plant performance and soil functions [11].

For degraded Cerrado soils, amendments featuring biomass residues (e.g., aquatic macrophytes, biochar, agro-industrial residues) efficiently enrich the soils with nutrients and organic matter [12–15]. These amendments enhance biological and physical soil conditions to allow faster recovery than unamended soils still lacking organic matter [16]. Besides, the residues selected (aquatic macrophytes and ash sugarcane bagasse) were chosen due to their abundance in the study region. Aquatic macrophytes cause problems in energy-generating in the hydroelectric power plants [17], and also the region has become a major producer of sugar cane, consequently, sugar, alcohol, and residues such as bagasse, cane straw, and bagasse ash, require an alternative for disposal [18]. Combining biomass residue amendments with the reintroduction of native vegetation, which add appropriate leaves, root biomass, and root exudates, further bolster soil functioning [19].

Abundance, diversity and biochemical attributes, and metabolic activities of microorganisms can serve as indicators of soil quality improvements [20], evidencing the success of restoration programs [21].

Other recovery indicators include the microbial quotient (*q*Mic), which defines the stock percentage of total organic carbon in the soil, and the metabolic quotient (*q*CO_2_), which shows specific respiration rates according to the CO_2_ released by microbial biomass as a function of time [22], it is expected that stressed soils present higher *q*CO_2_ values than less-stressed/natural soils [23]. The low stocks of organic compounds in degraded areas reveal corresponding low values of the microbial quotient [24]. In soils of preserved areas, that is to say, under native vegetation in the Cerrado, the values of *q*Mic range from 0.9 to 5.5 or 9 to 55% [25].

To develop tools for reconditioning these degraded areas stripped of topsoil, this work investigates whether native tree species combined with ash residue from sugarcane bagasse and/or aquatic macrophytes reestablish microbial activity and recover soil chemical properties in a severely degraded area, from where the surface horizons and native vegetation were removed and remained without vegetation cover since the 60s. We collected samples after 5 years of intervention and compared results with soils collected from an undisturbed Cerrado site as well as a degraded area without intervention.

Our hypothesis is that the addition of organic matter and nutrients, via regional residues, associated with soil tillage, can improve edaphic conditions of severely degraded soil and favor the re-establishment of vegetation and soil microbiota.

## Materials and methods

### Field trial and sampling

In November 2011, an experimental area of 3,4 ha for recovery was established at the Teaching, Research and Extension Farm of the São Paulo State University (UNESP) Ilha Solteira Campus, in Selvíria, state of Mato Grosso do Sul, Brazil (20° 22’ 22" S and 51° 24’ 59" W). This site remained without vegetation cover for 50 years since the construction of the Ilha Solteira Hydroelectric Power Plant removed up to 12 m of its topsoil.

**Fig 1:**
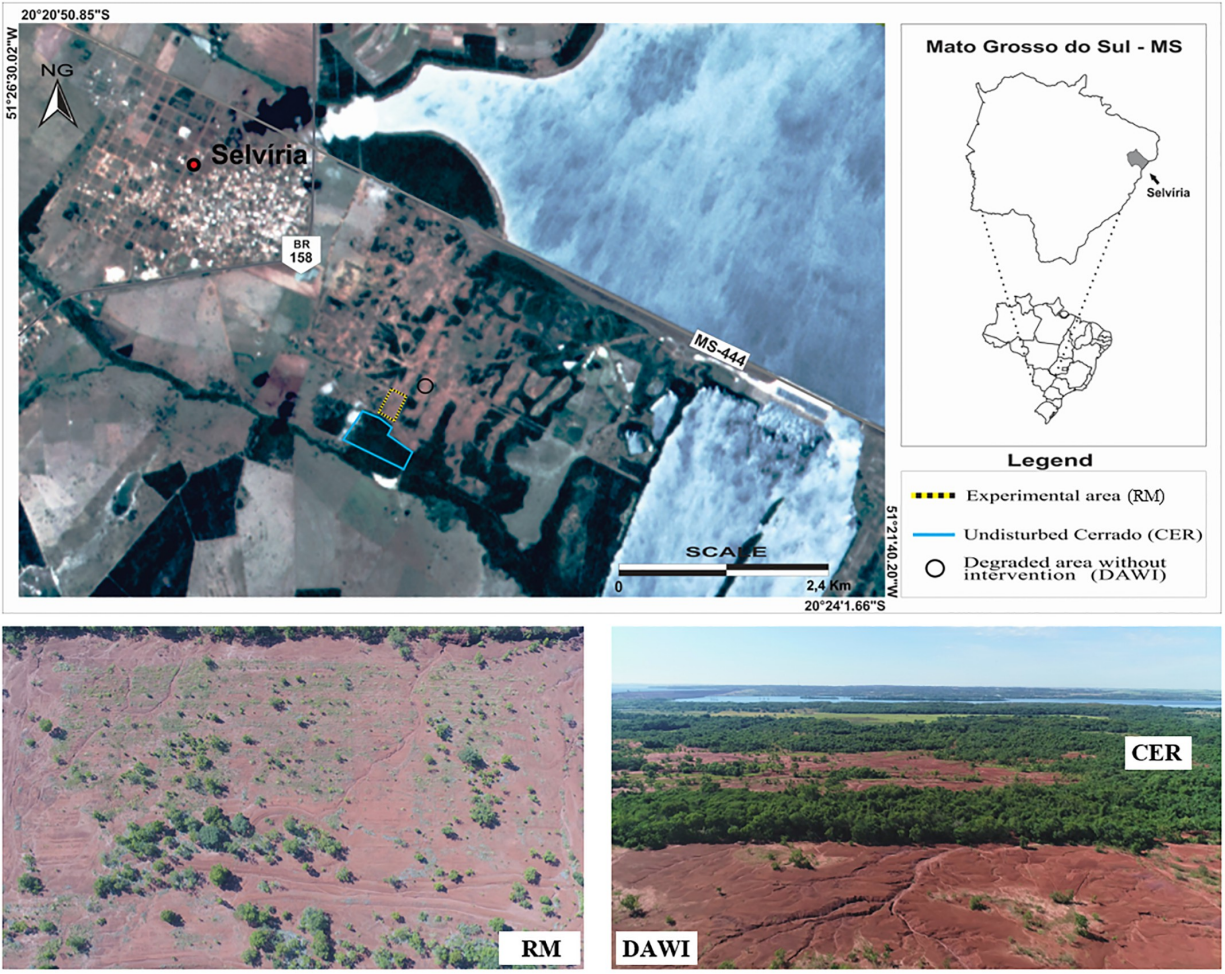
Aerial view of the research site illustrating the degraded area without intervention (DAWI, black circle), the area under restoration management (RM; dotted rectangle), and the undisturbed Cerrado (CER; solid blue polygon) in Selvíria, Mato Grosso State, Brazil.

**Fig 2:**
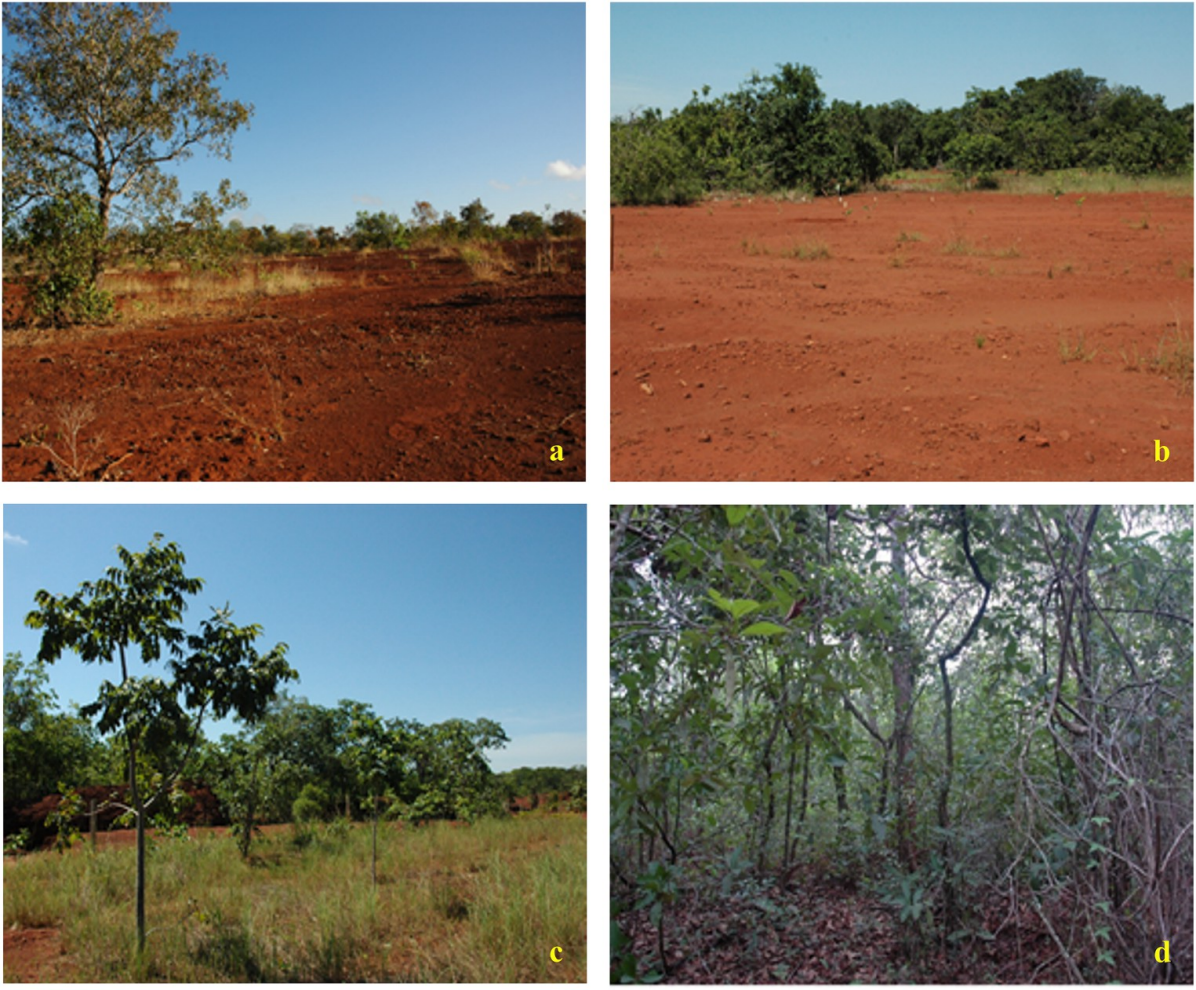
Experimental areas: (a) degraded area without intervention (DAWI), (b) AM00+BA00 receiving no amendments, (c) AM16+BA45 in 2016 after 5 years of soil conditioning treatments, and (d) undisturbed Cerrado (CER). (AM = aquatic macrophytes, BA = ash from sugar cane bagasse, applied at 00, 16 and 45 t ha^-1^).

The experimental design of the recovery management (RM) trial was randomized blocks composed of different rates of aquatic macrophytes and rates of sugar cane bagasse ash applied in strips, and using a 3 x 4 factorial scheme composed of 3 rates (0, 16 and 32 t ha^-1^) of aquatic macrophytes (AM, C:N ratio of 16.5) and 4 rates (0, 15, 30 e 45 t ha^-1^) of sugar cane bagasse ash (BA, C:N ratio of 93.4). The chemical characterization of the residues, AM and BA, are presented in S1 and S2 Tables. This factorial approach featured 12 treatments with 3 replicates each for a total of 36 plots with 600 m^2^ area per plot. To appraise the recovery progress, comparative analysis bracketed these interventions with similar evaluations of soil from an undisturbed Cerrado (CER) as well as a degraded area with no intervention (DAWI) (Figs 1 and 2). The granulometric analysis for these soil sites was obtained by the pipette method [26] (S3 Table). The amendment residues were collected locally. AM residues comprised a mixture of aquatic macrophyte species containing *Egeria densa* Planch., *Egeria najas* Planch., *Ceratophyllum demersum* L., *Eichhornia azurea* Kunth, *Eichhornia crassipes* (Mart.) Solms., *Pistia stratiotes* L. and *Typha latifolia* L., reported for the Jupiá Hydroelectric Power Plant in Três Lagoas/Brazil [27]. The ash was collected in the boiler at Alcoolvale: Sugar and Alcohol S.A., in Aparecida do Taboado/Brazil. Residues air-dried for 120 days before incorporation into the degraded soils.

**Fig 3:**
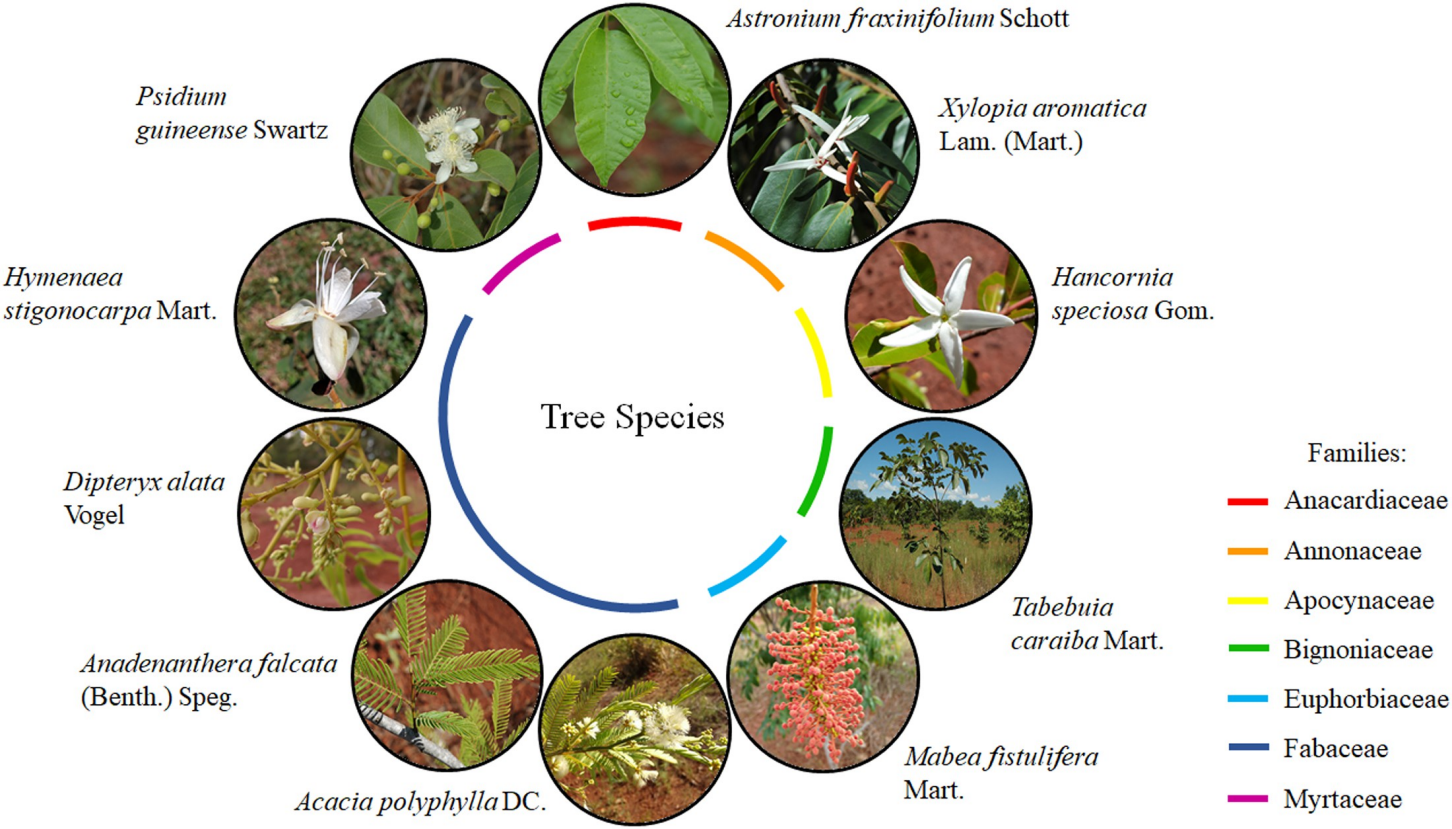
Native tree species introduced as seedlings to the restoration management (RM) area.

In February 2012, three months after soil residues incorporation, seedlings of 10 native species of Cerrado were introduced into the experimental area (Fig 3). Planting in pits of 0.40 m depth with 4.0 x 5.0 m spacing, each plot received three individuals of each species, totaling 1,080 seedlings.

After 5 years of intervention, the area was evaluated for soil fertility and microbiological activity. Soil samples collected during the rainy season (March 2016) at depths of 0.0 to 0.10 m in an assembled sample from 6 individual collections per plot in the experimental area (RM).

### Plant growth

Delineated in the field via tape measure at five years after planting, tree heights (cm) of the seedlings characterized each species’ survival.

Conducted during the rainy season (March 2016), the monitoring system for spontaneous vegetation canopy coverage in the RM treatments employed an inverted L-shaped stand to orient a camera parallel to the surface at a consistent 1.6 m height without casting shadows. In each of the 36 plots, images captured an area of approximately 2 m^2^ with 5 random repetitions. APS ASSESS 2.0 software estimated the percentage of ground vegetation cover (COVER) from the images [28].

### Soil chemistry

Quantifying soil exchangeable phosphorus (P), potassium (K), calcium (Ca), magnesium (Mg) and aluminum (Al) used ion exchange resins and Atomic Absorption Spectrophotometry—AAS [29, 30]. The total organic carbon (TOC) determination followed the Walkey-Black method with modifications [31]. This method uses sodium dichromate instead of potassium dichromate, due to the greater solubility of the former. In addition, the oxidation of organic matter is done cold, by simply stirring the soil in a solution containing sodium dichromate and sulfuric acid [32]. The pH was measured in water (dry soil: distilled water ratio of 1:2.5) [33] and total nitrogen (TN) by Kjeldahl method [34].

### Microbial-related soil properties

Soil respiration quantification relates the C-CO_2_ released by microbial respiration to the titration of a free base, NaOH to calculate of the amount of CO_2_ by subtraction [35]. First, 100 g of sampled soil was sieved (2 mm), weighed and placed in screw-cap glass jars along with a flask in the center containing 10 mL of 0.1 mol L^-1^ NaOH. With soil moisture corrected to 70%, the jars were then hermetically sealed. The incubation time was determined by a calibration curve developed by monitoring on alternate days. Titration of the free NaOH used HCl (0.1 mol L^-1^) and the phenolphthalein indicator (1%). As a control, glass jars were prepared, without soil, containing flasks with NaOH.

Microbial biomass carbon (MBC) quantification uses two 10 g samples of extracted soil per replicate and applies the fumigation-extraction method [36]. In this process, one sample is fumigated with chloroform, then both are analyzed by spectrophotometer read at a wavelength of 495 nm [37]. The carbon that settles with the death of the fumigated microorganisms allows a less turbid sample compared to the non-fumigated samples. The metabolic quotient (*q*CO_2_) represents the amount of C-CO_2_ released per MBC unit, estimated by the ratio of C-CO_2_ released/MBC, that is: μg C g^-1^ dry soil day / μg C g^-1^ dry soil, while the microbial quotient (*q*Mic) was calculated by the ratio between MBC and total organic carbon of the soil (TOC) expressed as a percentage [38].

### Data analysis

Analyses of variance (ANOVA) was employed to test the effects of soil amendments on plant growth, soil chemistry, and microbial-related soil properties. The analyses were performed by fitting the data into a linear mixed effect model using ‘lme’ function of the R software, considering blocks as a random factor. Before subjecting the data to ANOVA, the data homogeneity of variances (Levene’s test) and normality of the residuals (Shapiro-Wilk test) were tested. If the requirements for ANOVA were not met, data were log-transformed. Dunnett’s Multiple comparison post-hoc test was used to compare each experimental group with a control group. Dunnett’s test allowed us to identify significant changes by caused by the RM treatments compared to conditions in the site without any intervention (i.e., DAWI). We also compared each RM treatment with the conditions in the undisturbed site (i.e., CER), which reflect the improvement goals for vegetation, soil nutrients, and microbial activities for the sites under restoration. Hence, Dunnett’s mean test was used to compare each RM treatment between the reference sites, i.e., DAWI and CER (p <0.05) using “PMCMRplus” package in R. Tukey test compared means within the RM treatments (p <0.05) to identify what rate of inputs would be ideal to achieve changes in vegetation, soil nutrients, and microbial activities. For Tukey test, we used “multicomp” package in R. Pearson’s correlation analysis examined the relationship between plant, soil, and microbial variables using the “corrplot” package in R. All statistical analyses were performed in R 3.3.2 [39].

## Results

### Species survival and tree performance

**Fig 4:**
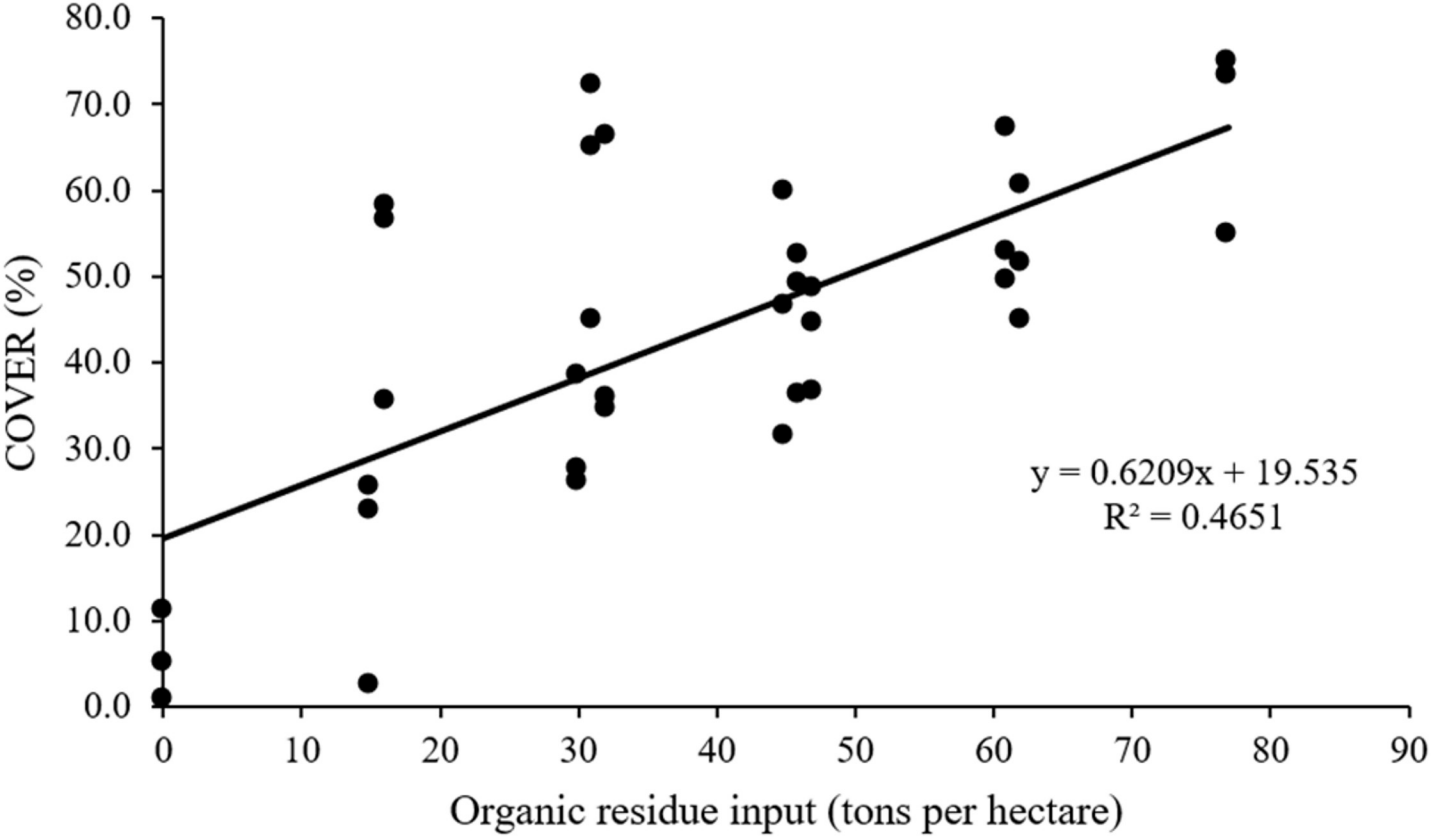
Correlation between ground vegetation cover (COVER) and residue inputs.

**Fig 5:**
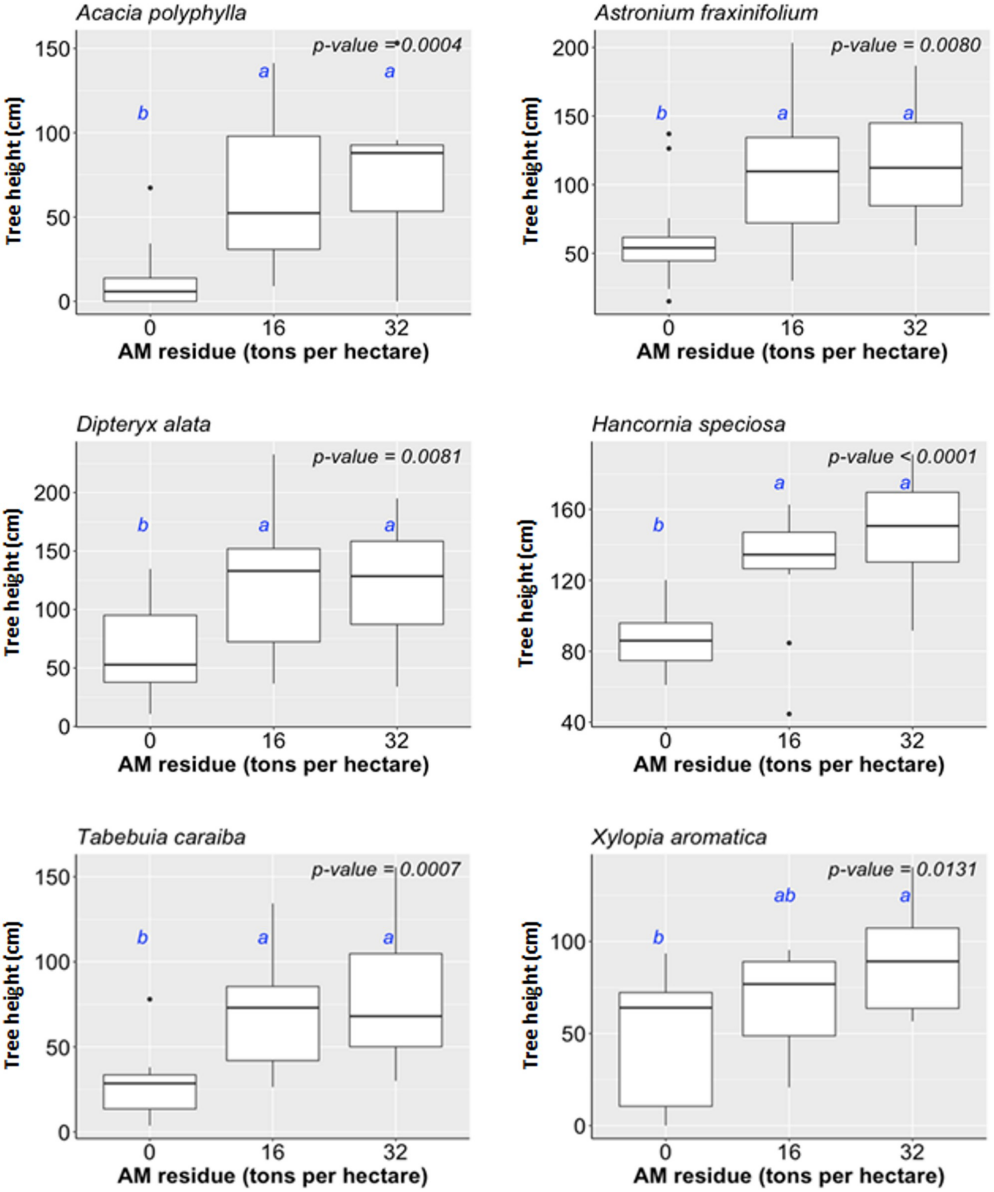
Influences of AM residues on tree height.

**Fig 6:**
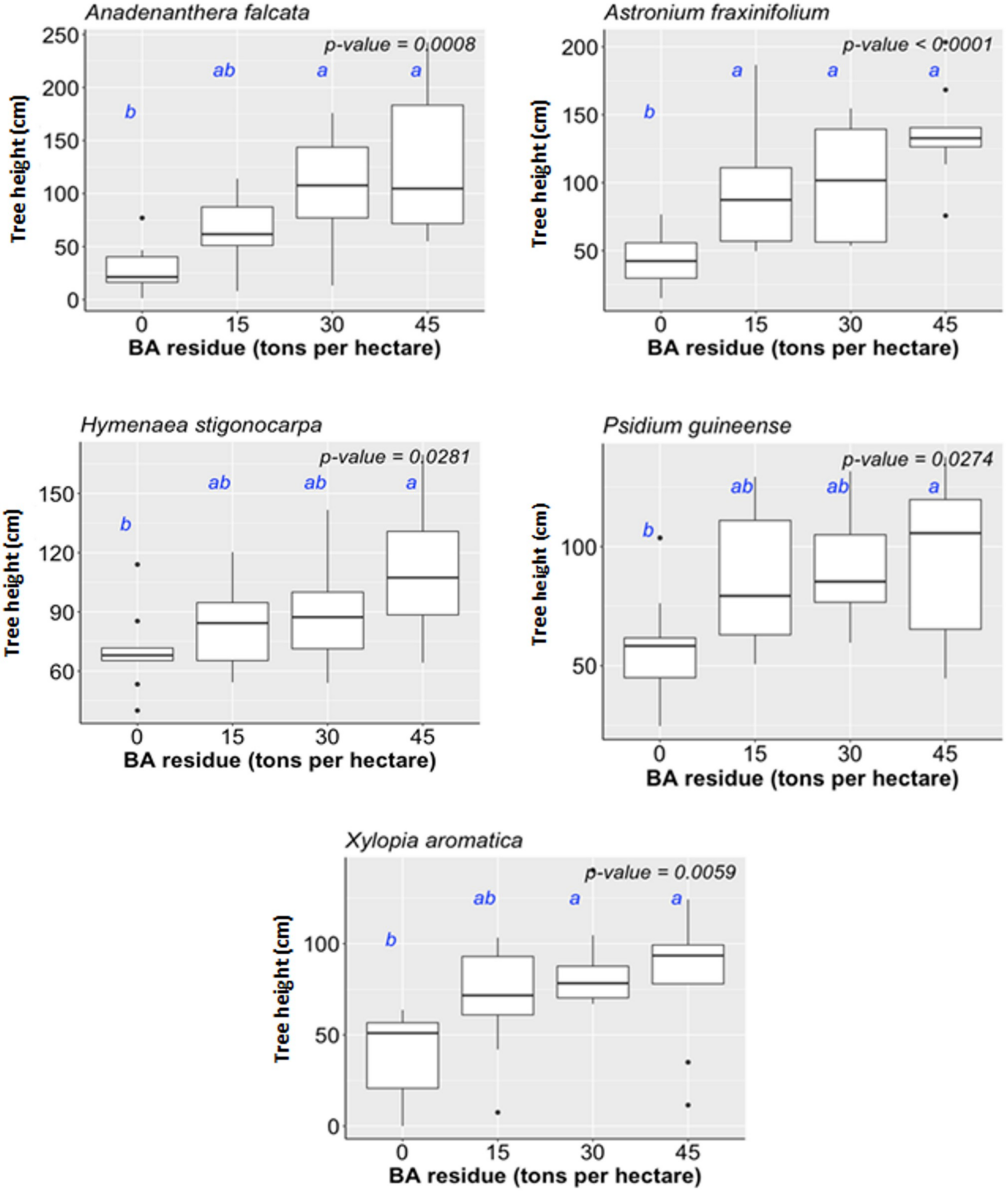
Influences of BA residues on tree height.

We estimated the percentage of ground vegetation cover (COVER) by spontaneously grown vegetation in the RM treatments (Tables 1 and 2). The species were a mix of herbaceous and shrub arboreal as well as native and alien origin. We observed a significant correlation between ground cover and biomass input, with the highest ground cover of 67.7% in AM32 + BA45 (r^2^ = 0.47; p<0.05; Fig 4). Plots without any input (i.e., AM00+BA00) achieved only 5.7% of the ground covered by vegetation. Tree survival proved consistent amongst all the RM treatments (p>0.05; 63,3 to 100%). There was no significant interaction effect of both amendments for tree height of any of the species (Table 1). AM residues, at either 16 and 32 tons per hectare, promoted tree height of *Acacia polyphylla*, *Astronium fraxinifolium*, *Dipteryx alata*, *Hancornia speciosa*, *Tabebuia caraiba and Xylopia aromatica*, by 489%, 78.2%, 93.2%, 57.6%, 177%, 57.5%, respectively, compared to their counterparts growing under AM00 (Fig 5). BA residues with incorporation rates larger than 15 tons per hectare, increased on average 174.7% the tree height of *A*. *fraxinifolium* compared to BA00 (Fig 6). BA residues with incorporation rates > 30 tons per hectare increased tree heights by 291% and 115% in *Anadenanthera falcata* and *X*. *aromatica*, respectively, compared to BA00. BA residues promoted height increases in *Hymenaea stigonocarpa* and *Psidium guineense*, but only at incorporation rates of 45 tons per hectare (56.4% and 65.6% increased, respectively, compared to BA00).

**Table 1:** Main and interactive effects of aquatic macrophyte (AM) residue, sugar cane bagasse ash (BA), their interaction (AMxBA), and of the three studied areas—Degraded area without intervention (DAWI), recovery management treatments (RM) and undisturbed Cerrado (CER), on vegetation, soil chemistry, and microbe-related soil properties.

Observed variables	AM	BA	AMxBA	Overall†
COVER	**	**	**	NA
*A*. *falcata*	ns	***	ns	NA
*P*. *guineense*	ns	*	ns	NA
*D*. *alata*	**	ns	ns	NA
*Mabea fistulifera*	ns	ns	ns	NA
*A*. *fraxinifolium*	**	***	ns	NA
*T*. *caraiba*	**	ns	ns	NA
*H*. *stigonocarpa*	ns	*	ns	NA
*H*. *speciosa*	***	ns	ns	NA
*A*. *polyphylla*	**	ns	ns	NA
*X*. *aromatica*	*	**	ns	NA
pH	**	**	*	*
TOC	ns	ns	ns	**
TN	ns	ns	ns	**
K	**	ns	ns	**
Ca	ns	*	*	**
Mg	ns	ns	ns	*
Al	ns	**	ns	**
Soil respiration	ns	ns	ns	*
MBC	ns	ns	ns	*
qCO2	ns	ns	ns	ns
qMic	ns	ns	ns	ns

*** p < 0.001,

** p < 0.01 and

* p < 0.05.

^†^ Analysis of variance (ANOVA) including DAWI, CER, and RM treatments.

NA: The analysis does not apply, as ground vegetation cover and tree height were not estimated in DAWI and CER.

**Table 2:** Mean values and standard deviation for ground vegetation cover (COVER) across the restoration management (RM) treatments. Mean followed by the same letter do not differ significantly by Tukey test (p <0.05).

RM treatments	Total biomass input (t ha^-1^)	COVER (%)
**AM00 + BA00**	0	5.7 ± 5.2 d
**AM00 + BA15**	15	16.8 ± 12.6 cd
**AM00 + BA30**	30	30.7 ± 6.9 bcd
**AM00 + BA45**	45	46.0 ± 14.2 abc
**AM16 + BA00**	16	50.1 ± 12.7 ab
**AM16 + BA15**	31	60.6 ± 14.0 ab
**AM16 + BA30**	46	45.9 ± 8.7 abc
**AM16 + BA45**	61	56.6 ± 9.4 ab
**AM32 + BA00**	32	45.6 ± 18.0 abc
**AM32 + BA15**	47	43.2 ± 6.1 abc
**AM32 + BA30**	62	52.4 ± 7.9 ab
**AM32 + BA45**	77	67.7 ± 11.3 a

### Soil chemical properties

Of the soil indicators measured, only soil pH, K, and Al responded to amendment inputs (Table 3). Treatments receiving the highest AM residue rate with any non-zero BA input (i.e., AM32+BA15, AM32+BA30, and AM32+BA45) increased 106% soil pH (5.2) and 277% K (0.9 mmol_c_ kg^-1^), compared to DAWI (pH = 4.8; K = 0.3 mmol_c_ kg^-1^). Only the RM treatments that included BA input reduced Al concentration, on average by decreasing 63,48% comparing to DAWI. Soil pH, K, Ca and Mg concentrations were inversely correlated with Al concentrations. Increases in soil pH and K concentrations were significantly correlated with ground vegetation cover and soil respiration. Compared to CER, the reference for an undisturbed Cerrado area, pH reached similar levels in treatments receiving 30 and 45 tons of BA per hectare or AM rates equal or above 16 tons of AM per hectare, with the exception of AM16 + BA00. Concentrations of K and Ca reached similar levels to those in CER only in the AM32+BA15 treatment.

**Table 3:** Mean values and standard deviation for soil pH, total organic carbon (TOC), total nitrogen (TN), phosphorus (P), potassium (K), calcium (Ca), magnesium (Mg), and aluminum (Al), in the degraded area without intervention (DAWI), restoration management treatments, and undisturbed Cerrado (CER).

Sites	pH^†^	TOC	TN	P	K	Ca^†^	Mg	Al
		- - (g kg^-1^) - -		(mg kg^-1^)	- - - - - - (mmol_c_ kg^-1^) - - - - - -			
**DAWI**	4.8 ± 0.06	4.6 ± 0.45	0.5 ± 0.19	0.9 ± 0.00	0.3 ± 0.10	0.9 ± 0.00	1.2 ± 0,58	6.9 ± 0.91
**AM00 + BA00**	4.6 ± 0.04 b	4.6 ± 0.18	0.6 ± 0.18	0.9 ± 0.00	0.3 ± 0.10	0.9 ± 0.00 b	0.9 ± 0.00	5.8 ± 1.65^Φ^
**AM00 + BA15**	4.8 ± 0.05 ab	4.6 ± 0.60	0.6 ± 0.24	0.9 ± 0.00	0.3 ± 0.15	2.5 ± 0.58 ab	1.8 ± 1.00	3.2 ± 0.01*
**AM00 + BA30**	5.0 ± 0.08 a^Φ^	4.6 ± 0.28	0.5 ± 0.21	0.9 ± 0.00	0.4 ± 0.06	2.5 ± 0.58 ab	1.8 ± 1.00	2.6 ± 1.21*
**AM00 + BA45**	5.0 ± 0.1 a^Φ^	5.1 ± 0.47	0.5 ± 0.12	0.9 ± 0.00	0.5 ± 0.15	4.0 ± 3.21 ab	3.7 ± 4.36^Φ^	2.6 ± 0.91*
**AM16 + BA00**	4.8 ± 0.08 ab	4.3 ± 0.44	0.4 ± 0.12	0.9 ± 0.00	0.4 ± 0.10	1.8 ± 1.00 ab	0.9 ± 0.00	5.3 ± 1.84^Φ^
**AM16 + BA15**	4.9 ± 0.12 ab^Φ^	4.5 ± 0.19	0.5 ± 0.08	0.9 ± 0.00	0.5 ± 0.17	1.8 ± 1.00 ab	1.5 ± 1.15	2.6 ± 0.91*
**AM16 + BA30**	5.1 ± 0.01* a^Φ^	4.6 ± 0.54	0.5 ± 0.23	0.9 ± 0.00	0.5 ± 0.06	2.8 ± 1.00 ab	1.8 ± 1.00	2.1 ± 0.91*
**AM16 + BA45**	5.0 ± 0.14 a^Φ^	5.1 ± 0.56	0.4 ± 0.11	0.9 ± 0.00	0.5 ± 0.15	1.8 ± 1.00 ab	1.2 ± 0.58	3.4 ± 0.46
**AM32 + BA00**	4.9 ± 0.15 ab^Φ^	4.3 ± 0.32	0.6 ± 0.15	0.9 ± 0.00	0.5 ± 0.23	2.2 ± 1.15 ab	1.5 ± 1.15	3.2 ± 0.01*
**AM32 + BA15**	5.2 ± 0.09* a^Φ^	4.2 ± 0.46	0.8 ± 0.31	0.9± 0.00	0.8 ± 0.17*^Φ^	4.6 ± 1.00 a^Φ^	2.5 ± 0.58	1.6 ± 0.8*
**AM32 + BA30**	5.1 ± 0.17* a^Φ^	5.15 ± 1.38	0.6 ± 0.19	0.9 ± 0.00	0.8 ± 0.12*	1.8 ± 0.00 ab	1.5 ± 0.58	2.6 ± 1.83*
**AM32 + BA45**	5.1 ± 0.22* a^Φ^	4.9 ± 0.61	0.5 ± 0.19	0.9 ± 0.00	0.9 ± 0.49*^Φ^	3.1 ± 2.31 ab	1.8 ± 1.00	2.1 ± 2.42*
**CER**	5.0 ± 0.14	12.8 ± 1.97*	1.5 ± 0.19*	7.1 ± 0.58*	1.2 ± 0.00*	9.30 ± 7.21*	6.80 ± 3.51*	7.1 ± 2.86

* = Mean values with asterisks are significantly different from control (DAWI) by the Dunnett test (p < 0.05).

^†^Significant interaction effect between AM and BA amendments. Means followed by the same letter, in the columns do not differ significantly by Tukey test (p <0.05).

^Φ^ Not significantly different than undisturbed Cerrado (CER)

AM = aquatic macrophytes applied at 0, 16, and 32 t ha^-1^.

**Fig 7:**
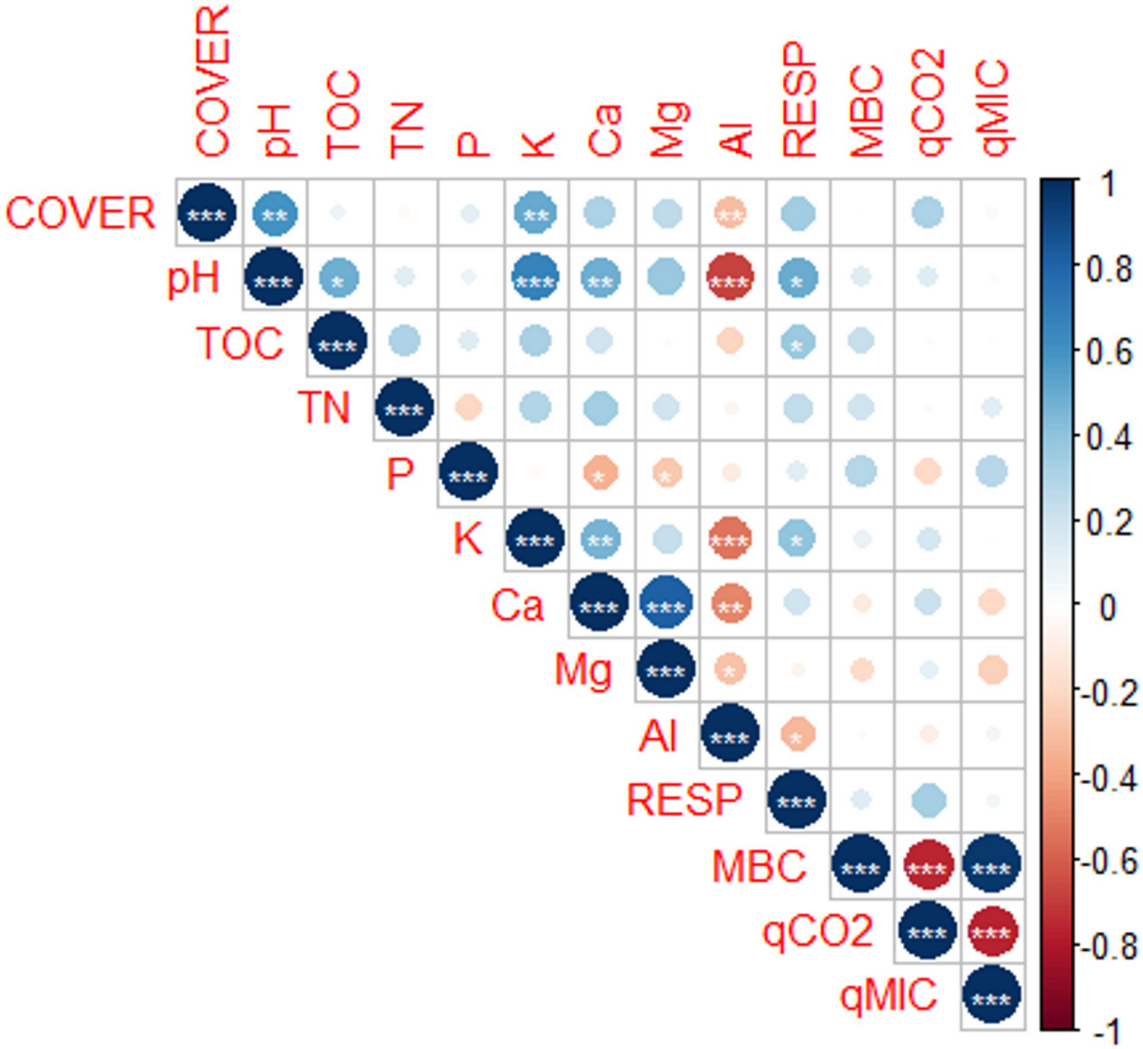
Correlation matrix among the variables soil cover by vegetation (COVER), pH, total organic carbon (TOC), total nitrogen (TN), phosphorus (P), potassium (K), calcium, magnesium (Mg), aluminum (Al), soil respiration (RESP), microbial biomass carbon (MBC), metabolic quotient (*q*CO2), and microbial quotient (*q*MIC) of the soil, across the restoration management treatments. ***, ** and * represent significant values for p ≤ 0.001, p ≤ 0.01, and p ≤ 0.05, respectively.

The correlation analysis (Fig 7) positively aligns TOC with N, P and Ca, particularly because they are found in the composition of organic matter, thus resulting in increases of these nutrients in the remaining subsoil that received both AM and BA.

### Microbial indicators

Across all sites, CER demonstrated the highest soil respiration being significantly larger than DAWI and the RM (Table 4). No significant differences arose between the RM treatments and DAWI. The average soil respiration in the RM treatments was about 2.7 times lower than in CER.

**Table 4:** Mean values and standard deviation for soil respiration (RESP), microbial biomass carbon (MBC), metabolic quotient (qCO_2_) and microbial quotient (qMic) inthe degraded area without intervention (DAWI), restoration management treatments, and undisturbed Cerrado (CER).

Site	RESP	MBC	*q*CO_2_	*q*Mic
	μg C g^-1^ dry soil day	mg C g^-1^ dry soil	#	(%)
**DAWI**	6.02 ± 2.08	0.11 ± 0.03	0.040 ± 0.002	2.55 ± 0.52
**AM00 + BA00**	3.05 ± 1.57	1.29 ± 0.48*	0.003 ± 0.002*^Φ^	28.30 ± 11.73*^Φ^
**AM00 + BA15**	6.51 ± 1.46	0.81 ± 0.21*	0.008 ± 0.002*^Φ^	19.56 ± 7.13^Φ^
**AM00 + BA30**	5.53 ± 3.68	1.20 ± 0.33*	0.007 ± 0.003*^Φ^	25.92 ± 6.62*^Φ^
**AM00 + BA45**	6.17 ± 3.10	0.69 ± 0.25*	0.009 ± 0.002*^Φ^	13.90 ± 6.14^Φ^
**AM16 + BA00**	5.62 ± 0.73	0.71 ± 0.50*	0.011 ± 0.008*^Φ^	16.12 ± 10.47^Φ^
**AM16 + BA15**	5.22 ± 1.61	1.70 ± 0.51*	0.004 ± 0.002*^Φ^	38.36 ± 12.92*^Φ^
**AM16 + BA30**	7.78 ± 1.93	0.87 ± 0.16*	0.009 ± 0.003*^Φ^	19.31 ± 4.78^Φ^
**AM16 + BA45**	8.41 ± 3.33	1.31 ± 0.70*	0.007 ± 0.001*^Φ^	25.28 ± 10.71*^Φ^
**AM32 + BA00**	5.53 ± 1.33	0.58 ± 0.26*	0.011 ± 0.005*^Φ^	13.28 ± 4.89^Φ^
**AM32 + BA15**	8.66 ± 2.99	1.91 ± 0.25*	0.005 ± 0.002*^Φ^	45.16 ± 4.37*^Φ^
**AM32 + BA30**	8.01 ± 1.91	1.70 ± 0.41*	0.005 ± 0.002*^Φ^	35.39 ± 14.29*^Φ^
**AM32 + BA45**	6.17 ± 0.91	0.73 ± 0.37*	0.010 ± 0.005*^Φ^	15.47 ± 9.12^Φ^
**CER**	17.67 ± 0.24*	3.47 ± 0.08*	0.005 ± 0.000*	27.58 ± 3.88*

* = Mean values with asterisks are significantly different from control (DAWI) by the Dunnett test (p < 0.05).

^Φ^ Not significantly different than undisturbed Cerrado (CER)

AM = aquatic macrophytes applied at 0, 16, and 32 t ha^-1^.

BA = ash bagasse residue applied at 0, 15, 30, 45 t ha^-1^.

^#^ = *q*CO_2_: μg C g^-1^ dry soil day / μg C g^-1^ dry soil.

The undifferentiated RM group MBC was a third of CER (p<0.05) and 10 times larger than DAWI. The proportion of MBC to TOC, represented by the microbial quotient (*q*Mic), ranged from 2–38% with the lowest in DAWI, the CER statistically inseparable from RM with AM16 + BA15 as the highest treatment. The microbial metabolic quotient (*q*CO_2_), which represents the respiration-to-MBC ratio, was the highest in the DAWI treatment and significantly larger than *q*CO_2_ in indistinguishable CER and RM treatments (Table 4).

It is noted that even in the treatment where only the subsoil turmoil occurred (AM00 + BA00) the BMC increased more than 1000% and reduced the *q*CO_2_ in 1200% compared to DAWI. In addition, 50% of the treatments, including the CER, presented some significant distance from the DAWI regarding the *q*Mic, where in mean these treatments increased 730% *q*Mic than DAWI.

Soil respiration correlated significantly with pH, TOC, and K. Soil respiration was also found to be inversely related to exchangeable aluminum content (Fig 7).

## Discussion

### Increasing soil pH while lowering aluminum concentration drives soil and vegetation improvements

In Oxisols, such as those that dominate Cerrado soils, pH_water_ and Al range from 4.0 to 5.3 and 1 to 9.3 mmol_c_ kg^-1^, respectively [15, 40–42]. Many Cerrado plants survive in soils with high Al content (13.73 mmol_c_ Al dm^-3^) [43], however, their growth can be limited by the low availability of essential nutrient cations such as ammonium, calcium, magnesium, and potassium [44]. Under acidic conditions, base cations become scarce as Al^+3^, H^+^, and Mn and Fe-bearing minerals prevail in nutrient solutions [45]. In this study, inputs of AM and BA increased soil pH and reduced Al concentration, and these changes aligned with observed increases in vegetation growth. Under the highest rates of input (AM 32 + BA 45), soil pH was able to reach CER levels, which suggests the system may be restoring. As expected under these more favorable conditions, AM 32 + BA 45 also showed significantly higher availability of the valuable cation K when compared to the degraded area without intervention (DAWI). Similarly, a study in degraded Oxisol showed that 21 months after fertilization with sheep increased soil pH from 4.2 to 4.6 and Al^+3^ decreased from 4.6 to 2.2 mmol_c_ dm^-3^, stimulating vegetation growth from 11.37 and to 48.15 g m^-2^ [46]. It is possible that vegetation growth did not result solely from the dissolution of base cations upon soil pH increase; the amount of nutrients added through the AM and BA amendments was very high. Previous research proposes N concentration (%) as the first determinant of N release, where resources with N concentration < 2.5% can release about 40% of their total amount [47]. P and K-rich plant based organic amendments can mineralize up to 80 and 100% of their P and K contents within three months of incorporation [48]. Thus, based on amendment input concentrations, the AM 32 + BA 45 treatment added 837, 93, and 215 kg ha^-1^, of N, P, and K respectively.

Indeed, ground vegetation cover increased linearly with biomass input, from 5.7% (AM 00 + BA 00) to 67.7% (AM 32 + BA45) of ground cover. This aligns with several studies demonstrating the potential of soil amendments and biomass inputs for restoring degraded areas [22, 49–52]. Biomass inputs denote nutrient inputs which propel vegetation growth and favors the appearance of spontaneous vegetation that combat soil erosion [53, 54], reduce soil temperature [55, 56], and increase biological activity [57]. It was demonstrated that when using biochar, biosolids, wood chips, singly or combined, in post-mine sites, soil properties improved resulting in increased nutrient availability, soil moisture, and consequently elevated plant cover from 17% (Control) to 58% (Biosolid + biochar + wood chip treatment) [58].

Henceforth, this study demonstrates how a gradient of AM and BA amendments span the range from DAWI to CER in pH and Aluminum concentration can release essential cations that fuel the vegetative growth valuable to ecosystem stability.

### Microbial improvements associated to the residues

In addition to vegetative biomass and soil nutrients, microbial communities can reveal the re-establishment of vital functions in systems under restoration, as they respond quite rapidly to the addition of organic materials and carry out many relevant ecosystem processes such as nutrient cycling [24, 59].

For this study, the microbially-based indicators needed to be sensitive to inputs of organic materials as well as cost-effective considering the sample size. All restoration management treatments (RM) showed, on average, an order of magnitude more microbial biomass carbon (MBC) than DAWI, which neglected organic residue input, tillage, or tree seedling transplants. This also includes the AM00 + BA00 site, which accounted with only tillage and tree seedling transplants. The decrease in the microbial metabolic quotient (respiration-to-biomass ratio), or *q*CO_2_, under RM compared to DAWI indicates that RM microbial communities released less CO_2_ per unit microbial biomass than in DAWI [60]. The opposed the trend for *q*Mic, the proportion of MBC to total organic carbon. The observed *q*Mic increased in the RM sites, relative to DAWI, indicating a higher proportion of microbial carbon in the total carbon pool [61, 62]. Similar values were found among all RM treatments and CER for *qCO*_*2*_ and *qMic*, showing that remediation is producing conditions similar to our ideal undisturbed site. The quotients present specific respiration rates (*q*CO_2_) and stock of carbon in the soil (*qMic)*, the highest values of *q*Mic indicate the maintenance of carbon in the soil [61], revealing that after five years of treatments, the RM soils show positive changes in microbial activity comparable to the specific respiration and stock of carbon from the CER. These microbial responses are expected due to the input of organic amendments and the physical changes caused by tillage [60, 63]. The organic compounds added in this trial provide energy and nutrients [19], and soil mechanical tillage in degraded soils decompact the surface to produce changes in the physical and mechanical properties of soil that improve water infiltration and aeration [64].

Five years later, it is still possible to see the beneficial responses from the RM. Evaluating a soil restoration experiment, using biosolids and nutrients after the mechanical subsoiling, in Brazilian Federal District mining sites, was observed soil carbon continues to increase even after 9 years since the introduction of the amendments [65]. Thus, lasting soil productivity can be increased by the addition of natural amendments that stimulate the microbial activity to provide the nutrients and organic carbon to the soil. After vegetation establishes, the cycling of nutrients begin that maintain the activity of microorganisms for the long-term [19, 66]. We see the formation of this soil fertility in this study, as both soil respiration and MBC correlated significantly with TOC, N and P, K, Ca and Mg, suggesting that the microbial activities are helping to maintain TOC and nutrient availability in the soil [59].

### Plants responses to residues inputs and soil improvements

Using only plants native to Cerrado and occurring in the central Brazil, all 10 species survived the experiment. Some plant heights responded to the AM residues and others responded specific to BA residues, however the combination of the amendments promoted no additional effects on tree height. These responses can be related to the improvements in soil nutrients, soil coverage and consequently in C cycle. Soil nutrients are decisive for the successful reestablishment and growth of plants [67, 68], and several soil conditioners and fertilizers improve soil nutrients stimulating recovery in degraded areas [22, 52]. Only *Mabea fistulifera* did not respond to the amendments added, which may indicate that the species is adapted to adverse conditions.

Other studies verified the effects of organic amendments to the growth of Cerrado plants. Biochar and cattle manure combined in different doses promote development of seedlings of *Magonia pubescens*, a native Cerrado species. The larger doses of combined manure and biochar (CM30% + BC30%) presented better results for average height, differing from the control with 9% increase in height (2.2 cm) [69]. Different doses of agro-industrial residues (ash) in *Hymenaea stigonocarpa* produced positive effects after 8 months, such as a linear increase of height, ultimately yielding approximately 24% compared to control [13].

The species, *Astronium fraxinifolium*, augmented height incrementally as the doses of both residues increased (Figs 5 and 6), proving to be a good alternative for recovery experiments using soil amendments.

## Conclusions

The combination of the amendments (aquatic macrophytes and ash from sugarcane bagasse) increased the pH and reduced the Al, consequently increasing the availability of nutrients, such as K. The improvements in soil quality prompted vegetation growth, evidenced by increases in the biomass of spontaneous plants and the height of native tree species. The restoration management treatments also boosted soil biological activities.

Comparing to the degraded area without intervention, the restoration management treatments increased microbial biomass-C. And contrasted to undisturbed Cerrado, showed similar results to *q*MIC and *q*CO_2_.

After 5 years of intervention, the restoration management treatments, which include biomass residues and the reintroduction of native vegetation, can effectively rehabilitate an intensively degraded area such as those of exposed sub-soils in the Brazilian Cerrado.

## Supporting information

S1 Table Composition of aquatic macrophytes residue. (DOCX)

S2 Table Physical and chemical characterization of ash from sugarcane bagasse residue. (DOCX)

S3 Table Soil granulometry and texture of the study areas. (DOCX)

S1 Fig Restoration management area croqui. (DOCX)

S1 Data (XLSX)
