# Supplementary material for: Biomass residues improve soil chemical and biological properties reestablishing native species in an exposed subsoil in Brazilian Cerrado
Source: PLoS One. 2022 Jun 27;17(6):e0270215. doi: 10.1371/journal.pone.0270215 (PMC9236270; doi:10.1371/journal.pone.0270215)
Supplement: S1 Table — (DOCX) [file pone.0270215.s001.docx]

**S1 Table.** Composition of aquatic macrophytes residue.

| Aquatic Macrophytes | | |
| --- | --- | --- |
| Parameter | Unit | Value |
| Boron | mg kg^-1^ | 27 |
| Calcium | g kg^-1^ | 11.6 |
| Copper | mg kg^-1^ | 57 |
| Iron | mg kg^-1^ | 2000 |
| Magnesium | g kg^-1^ | 2.4 |
| Manganese | mg kg^-1^ | 194 |
| Nitrogen | g kg^-1^ | 17.6 |
| Phosphorus | g kg^-1^ | 1.7 |
| Potassium | g kg^-1^ | 6.5 |
| Sulfur | g kg^-1^ | 6.7 |
| Zinc | mg kg^-1^ | 34 |
