# Supplementary material for: Biomass residues improve soil chemical and biological properties reestablishing native species in an exposed subsoil in Brazilian Cerrado
Source: PLoS One. 2022 Jun 27;17(6):e0270215. doi: 10.1371/journal.pone.0270215 (PMC9236270; doi:10.1371/journal.pone.0270215)
Supplement: S2 Table — (DOCX) [file pone.0270215.s002.docx]

**S2 Table.** Physical and chemical characterization of ash from sugarcane bagasse residue.

| Sugarcane Bagasse Ash | | |
| --- | --- | --- |
| Parameter | Unit | Value |
| Aluminum | mg kg^-1^ | 1710 |
| Arsenio | mg kg^-1^ | <1.0 |
| Barium | mg kg^-1^ | 25.7 |
| Boron | mg kg^-1^ | <3.2 |
| Cadmium | mg kg^-1^ | <0.4 |
| Calcium | g kg^-1^ | 5.3 |
| Chrome | mg kg^-1^ | 12.5 |
| Copper | mg kg^-1^ | 21.1 |
| Humidity (60-65 ° C) | % (m/m) | 3.2 |
| Iron | mg kg^-1^ | 1540 |
| Lead | mg kg^-1^ | 3.4 |
| Magnesium | g kg^-1^ | 1.1 |
| Manganese | mg kg^-1^ | 182 |
| Mercury | mg kg^-1^ | <1.0 |
| Molybdenum | mg kg^-1^ | <0.9 |
| N Ammoniacal | mg kg^-1^ | 220 |
| N Kejldahl | g kg^-1^ | 6.1 |
| N Nitrate-Nitrite | mg kg^-1^ | 421 |
| Nickel | mg kg^-1^ | <2.4 |
| Organic Carbon | g kg^-1^ | 570 |
| pH (H_2_0 1:10) | - | 5.2 |
| Phosphorus | g kg^-1^ | 0.86 |
| Potassium | mg kg^-1^ | 1617 |
| Selenium | mg kg^-1^ | <1.0 |
| Sodium | mg kg^-1^ | 18.0 |
| Sulfur | g kg^-1^ | 0.24 |
| Total solids | % (m/m) | 95.2 |
| Volatile solids | % (m/m) | 48.7 |
| Zinc | mg kg^-1^ | 12.4 |
