# Supplementary material for: Biomass residues improve soil chemical and biological properties reestablishing native species in an exposed subsoil in Brazilian Cerrado
Source: PLoS One. 2022 Jun 27;17(6):e0270215. doi: 10.1371/journal.pone.0270215 (PMC9236270; doi:10.1371/journal.pone.0270215)
Supplement: S3 Table — (DOCX) [file pone.0270215.s003.docx]

**S3 Table.** Soil granulometry and texture of the study areas.

| Site | Clay | Sand | Silt | Texture |
| --- | --- | --- | --- | --- |
|  | - - - - - - - - % - - - - - - - | | |  |
| RM | 48.1 | 40.9 | 11.0 | Clay |
| DAWI | 45.2 | 40.4 | 14.4 | Clay |
| CER | 30.9 | 58.1 | 11.0 | Sandy Clay Loam |

Restoration management treatments (RM), Degraded area without intervention (DAWI), and Undisturbed cerrado (CER).
