# Supplementary figures and images for: Biomass residues improve soil chemical and biological properties reestablishing native species in an exposed subsoil in Brazilian Cerrado
Source: PLoS One. 2022 Jun 27;17(6):e0270215. doi: 10.1371/journal.pone.0270215 (PMC9236270; doi:10.1371/journal.pone.0270215)

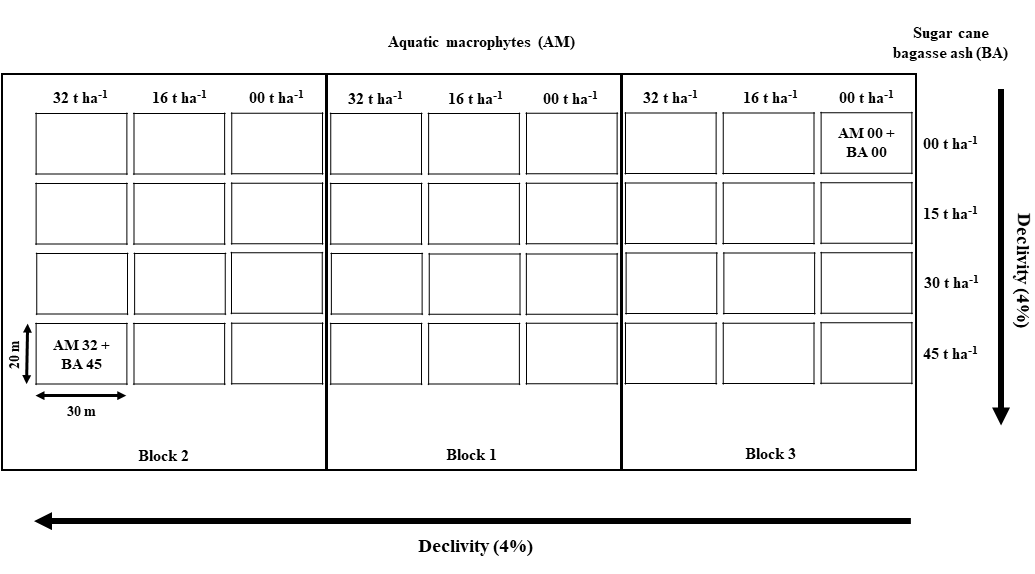

Supplement: S1 Fig — (DOCX) [file pone.0270215.s004.docx]
